# Behavioral and biological alterations following transplantation of ASD-associated gut microbiota in mice

**DOI:** 10.7717/peerj.20951

**Published:** 2026-03-24

**Authors:** Yang Gao, Dan Yang, Danni Wang, Rena Maimaiti

**Affiliations:** The First Affiliated Hospital, Xinjiang Medical University, Urumqi, China

**Keywords:** Autism spectrum disorder, Typically developing, Fecal microbiota transplantation, Gut microbiota

## Abstract

**Background:**

Alterations in the gut microbiota have been increasingly reported in individuals with autism spectrum disorder (ASD). However, the extent to which ASD-associated microbial communities are linked to behavioral and host biological changes remains to be clarified.

**Methods:**

Fecal microbiota transplantation (FMT) was performed using samples from children with ASD or typically developing (TD) controls into antibiotic-pretreated mice. Behavioral performance was evaluated using open field, social interaction, and spatial learning paradigms. Gut microbial composition was assessed by 16S rRNA gene sequencing. Peripheral immune-related parameters were assessed, including circulating regulatory T cells and serum cytokine profiles. Histological and molecular analyses were conducted in the terminal ileum and hippocampus, including immunohistochemistry, Western blotting, exploratory brain proteomics, and targeted qRT-PCR validation.

**Results:**

Mice receiving ASD-associated microbiota displayed altered behavioral performance, including reduced sociability and impaired spatial learning. 16S rRNA sequencing revealed distinct gut microbial profiles between ASD-FMT and TD-FMT groups, with increased abundance of Enterobacteriaceae and related taxa in ASD-FMT mice. Peripheral analysis showed reduced regulatory T cell levels and altered serum cytokine profiles. Histological examination identified changes in intestinal architecture and increased expression of glial markers in the hippocampus. Proteomic analysis indicated differential expression of proteins enriched in pathways related to neural structural organization and metabolic processes.

**Conclusions:**

These findings indicate that gut microbiota derived from children with ASD is associated with coordinated behavioral, peripheral immune-related, and brain histological changes in mice. This study provides integrative evidence supporting associations between ASD-associated microbial features and coordinated behavioral and biological alterations in the host.

## Introduction

Autism spectrum disorder (ASD) is a neurodevelopmental condition characterized by persistent difficulties in social communication, alongside restricted and repetitive patterns of behavior and interests. Over the past two decades, the reported prevalence of ASD has increased substantially, rising from approximately 1 in 150 children in 2000 to 1 in 36 among 8-year-old children in recent surveillance data (Maenner et al., 2023). Despite extensive research, the etiology of ASD remains incompletely understood and is widely considered to involve complex interactions between genetic susceptibility and environmental influences (Love et al., 2024).

In recent years, increasing attention has been directed toward the potential role of the gut microbiota in neurodevelopmental disorders, including ASD. The gut microbiota, often described as a functional “microbial organ” (Cristofori et al., 2021; Generoso et al., 2021) communicates with the central nervous system through multiple pathways, including microbial metabolites, immune signaling, and neuroendocrine mechanisms (Mirzaei et al., 2021; Needham et al., 2021). Clinical studies have consistently reported alterations in gut microbial composition in individuals with ASD, and several reports suggest associations between reduced microbial diversity and the severity of ASD-related behavioral features (Feng et al., 2024; Ronan, Yeasin & Claud, 2021). Accumulating evidence has shown that alterations in the gut microbiota can influence host behavior through multiple mechanisms, including immune modulation, microbial metabolites, and neuroimmune interactions. Building on these findings, the present study integrates behavioral, immunological, and neurobiological analyses to further examine microbiota–brain interactions.

Consistent with observations from clinical studies, animal models have provided important experimental support for a potential link between gut microbiota and behavior. In particular, fecal microbiota transplantation (FMT) from individuals with ASD into germ-free or antibiotic-treated mice has been shown to induce alterations in social behavior, repetitive activity, and learning-related tasks (Xiao et al., 2021). While these studies suggest that ASD-associated microbiota can influence host behavior, most have focused primarily on behavioral phenotypes, with limited integration of immune and neurobiological endpoints. Moreover, the extent to which immune regulation mediates microbiota–brain interactions in these models remains insufficiently explored.

Immune dysregulation has been repeatedly implicated in ASD pathophysiology. Individuals with ASD often exhibit elevated levels of pro-inflammatory cytokines in peripheral blood and brain tissue, along with alterations in immune regulatory pathways (Moreno, Abu Amara & Ashwood, 2025). Regulatory T cells (Tregs) play a critical role in maintaining immune homeostasis through suppression of excessive inflammatory responses and secretion of anti-inflammatory cytokines such as interleukin-10. Dysregulation of Treg number or function may contribute to a chronic low-grade inflammatory state, which has been proposed to influence blood–brain barrier integrity, microglial activation, and neurodevelopmental processes (She et al., 2026). Nevertheless, how gut microbiota alterations may modulate Treg-mediated immune responses in the context of ASD remains an open question.

Based on these considerations, the present study employed fecal microbiota transplantation from children with ASD and typically developing controls into antibiotic-pretreated mice to investigate microbiota-associated behavioral, immunological, and neurobiological changes. Using a multidimensional approach, we assessed behavioral performance, gut microbiota composition, peripheral immune profiles, intestinal and hippocampal histopathology, glial cell morphology, and exploratory brain proteomic alterations. Rather than establishing causality, this study aims to characterize associations across the gut–immune–brain axis in an experimental FMT model, thereby providing integrative evidence to support future mechanistic investigations into microbiota-related pathways relevant to ASD.

## Materials & Methods

### Human participants

Healthy children (*n* = 5) undergoing routine health check-ups and children with newly diagnosed autism spectrum disorder (ASD) (*n* = 5) were recruited from the Department of Pediatrics at the First Affiliated Hospital of Xinjiang Medical University. All ASD diagnoses were made by experienced pediatric neurologists according to the DSM-5 diagnostic criteria. The participants were boys aged 3–5 years. Detailed inclusion and exclusion criteria, as well as demographic characteristics of both groups, are provided in Supplementary Materials 1 and 2. This study was approved by the Ethics Committee of the First Affiliated Hospital of Xinjiang Medical University (Ethical Approval No. K202506-37), and written informed consent was obtained from all participants.

### Fecal sample processing

For each group (ASD or healthy controls), fecal samples from individual participants were pooled within the same group, homogenized, and processed as described below. The samples were homogenized and diluted in sterile PBS (five mL/g), vortexed until thoroughly mixed, and then filtered through sterile gauze to remove large particulate matter. The suspension was centrifuged at 500 × g for 5 min at 4 °C, and the supernatant was collected. The supernatant was mixed with an equal volume of 50% sterile glycerol to achieve a final concentration of 25%, thoroughly mixed, aliquoted, and rapidly frozen. The samples were stored at −80 °C for FMT treatment.

### Animals

A total of 16 three-week-old male C57BL/6 mice (12–15 g) were obtained from the Animal Experimental Center of Xinjiang Medical University and randomly assigned to two groups (ASD-FMT, *n* = 8; TD-FMT, *n* = 8). The sample size was determined based on previous fecal microbiota transplantation studies using comparable behavioral, immunological, and histological endpoints (Li et al., 2024; Yang et al., 2022), and was considered sufficient to detect group differences while minimizing animal use in accordance with ethical principles.

Mice were maintained under specific pathogen-free (SPF) conditions at 24 ± 1 °C, 50–70% relative humidity, and a 12-hour light/dark cycle (lights on from 8:00 AM to 8:00 PM). Animals were housed four per cage and provided with sterilized standard chow and autoclaved drinking water ad libitum.

Experimental interventions included pretreatment with a broad-spectrum antibiotic cocktail followed by fecal microbiota transplantation (FMT) from either children with ASD or typically developing controls. Behavioral tests were conducted at 6 weeks of age. No analgesics or anesthetics were administered during behavioral testing, as these procedures are non-invasive and do not induce pain or distress.

At the end of the experiment, animals were euthanized with an overdose of sodium pentobarbital under deep anesthesia. Death was confirmed by the absence of heartbeat and respiration together with fixed, dilated pupils. All tissues and blood samples were collected immediately under deep anesthesia, ensuring minimal post-mortem delay. Pentobarbital was only used for terminal procedures, and no analgesics or anesthetics were administered during behavioral testing. Although some euthanasia agents can modulate certain biomarkers, intraperitoneal pentobarbital at euthanasia doses has minimal impact on the key post-mortem measures used in this study and is not expected to confound the results (Mohamed et al., 2020; Tobar Leitão et al., 2021). Humane endpoints were predefined as >20% body weight loss, severe lethargy, or inability to access food or water; none of the animals met these criteria, and all animals were euthanized at the planned experimental endpoint. All procedures complied with the Chinese Guidelines for the Care and Use of Laboratory Animals and were approved by the Ethics Committee of Xinjiang Medical University (Approval No. IACUC-JT-20250114-06).

### Experimental timeline

Mice were housed in an SPF facility and allowed to acclimate for one week starting at 3 weeks of age. At 4 weeks of age, mice received a broad-spectrum antibiotic cocktail by oral gavage for five consecutive days to disrupt the endogenous gut microbiota. Fecal samples were collected immediately after antibiotic treatment to assess microbial depletion by measuring total fecal DNA. At 5 weeks of age, mice received fecal microbiota transplantation (FMT) by oral gavage. Behavioral testing was conducted at 6 weeks of age.

### Antibiotic treatment & fecal microbiota transplantation

To reduce the influence of the native gut microbiota prior to fecal microbiota transplantation, all mice were gavaged with a mixture of broad-spectrum antibiotics (vancomycin (50 mg/kg, CAS: 123409-00-7, MP Bio, California, USA), neomycin (100 mg/kg, CAS: 1405-10-3, MP Bio), penicillin (100 mg/kg, CAS: 69-52-3, MP Bio), and metronidazole (100 mg/kg, CAS: 443-48-1, MCE, New Jersey, USA) mixed in sterile). The treatment was administered twice daily for five consecutive days, with a gavage volume of 10 mL/kg (Li et al., 2020; Olson et al., 2018). The antibiotic mixture was freshly prepared daily.

After 5 days of antibiotic treatment, C57BL/6 mice (fecal samples from the mice after 5 days of gavage were compared with fecal samples from normal mice for genomic DNA analysis, as detailed in Supplement 3) were randomly divided into two groups (eight mice per group). These groups received fecal suspensions from ASD children (ASD + FMT (Autism Spectrum Disorder + Fecal Microbiota Transplantation), ASD-FMT) and healthy children (TD + FMT (Typically Developing + Fecal Microbiota Transplantation), TD-FMT). The gavage volume was 10 mL/kg, administered once daily for seven consecutive days (weight changes of mice during the fecal microbiota transplantation process are shown in Supplement 3).

### Behavioral testing

Behavioral tests were conducted on all experimental mice at 6 weeks of age, between 9:00 AM and 5:00 PM, with at least a 24-hour interval between each test. The following tests were performed in this order: open field test, three-chamber social interaction test, Y-maze, and water maze. All apparatus were cleaned with 75% ethanol between trials to eliminate olfactory cues. Behavioral tests and image analyses were performed by investigators blinded to group allocation.

**1. Open Field Test** (Software: digbehv4.5, Shanghai): Mice were placed in the center of the open field (40  × 40 cm), and the test duration was 5 min. The time spent by the mice in different regions (center, corner, and side) was recorded.

**2. Three-Chamber Social Interaction Test** (Software: digbehv4.5, Shanghai): This test was conducted in a 60  × 45  × 22 cm preference box, which includes a left chamber, a right chamber, and a central chamber (mice were placed in the central chamber to begin with to avoid any bias in the left–right chamber entry sequence). Before the official test, each mouse was allowed to freely explore the central chamber for 5 min. After the exploration period, a same-aged, same-sex unfamiliar mouse (Stranger 1) was placed in the left chamber, and the test mouse was observed for 10 min. The activity within the three chambers was recorded. The cage was then cleaned with 75% ethanol solution to remove feces and odors, and a second same-aged, same-sex unfamiliar mouse (Stranger 2) was placed in the right chamber, while Stranger 1 remained in the left chamber. The test mouse was observed for another 10 min, and the activity within the chambers was recorded. The contact time of the test mouse with the left (L) and right (R) chambers was measured (Rein, Ma & Yan, 2020).

**3. Y-Maze Working Memory Test** (Software: digbehv4.5, Shanghai): The Y-maze arms had a length of 50 cm, a width of 15 cm, and a height of 25 cm. Three arms were randomly designated as A, B, and C. Each test mouse was randomly placed at the end of one of the arms and allowed to explore the maze for 8 min. The sequence in which the mouse entered each arm was recorded. A valid entry was defined as the mouse exploring beyond two-thirds length of the arm. A spontaneous alternation was defined as a sequence of three different arm entries (*e.g.*, ABC, ACB, CAB, *etc.*). The total number of arm entries and the number of spontaneous alternations were recorded, and the spontaneous alternation rate (%) was calculated using the formula:

Spontaneous alternation rate (%) = (Number of spontaneous alternations/Theoretical maximum number of alternations (*i.e.*, total arm entries – 2)) ×100%.

**4. Water Maze Experiment** (WMT-100, Chengdu Techman Software Co., Ltd, Chengdu): The Morris water maze (diameter: 1.2 m, depth: 0.5 m) was used to assess the spatial learning and memory capabilities of the mice. This test consists of two stages:

**I. Navigation Test**:

**1. Environmental Adaptation**: On the first day, the platform was placed one cm below the water surface, and the mouse was allowed to swim freely for 120 s.

**2. Training Setup**: Clean water was filled in the pool, and the platform was placed one cm below the water surface in any quadrant. The water temperature was maintained at approximately 24 °C. Training was conducted for five consecutive days, with four training sessions per day (with entry from different quadrants). The training duration was fixed. During each session, the mouse was placed with its back to the platform, and the time spent searching for the platform was recorded. If the mouse successfully found the platform, it was allowed to stay there for 30 s; if not, it was guided to the platform and allowed to stay for 30 s. The escape latency (*i.e.,* the time taken to find the platform) was recorded.

**II. Spatial Exploration Test** (Day 6):

1. The platform was removed from the water, and the mouse was placed in a quadrant that was not the original platform location (all mice entered the water from the same location).

2. The following parameters were recorded during a 60-second test: time spent in the original platform quadrant and the number of crossings over the original platform location (Vorhees & Williams, 2006). (Statistical analysis of behavioral test parameters for both ASD-FMT and TD-FMT groups is presented in Supplement 4).

### 16S rRNA gene extraction, amplification, and sequencing

Fecal samples from TD-FMT and ASD-FMT mice were collected and stored at −80 °C. Genomic DNA was extracted using the TIANamp Stool DNA Kit (DP328, TIANGEN, China). DNA concentration and purity were assessed using a NanoDrop spectrophotometer and Qubit 3.0 Fluorometer, and DNA integrity was confirmed by agarose gel electrophoresis.

The V3–V4 region of the bacterial 16S rRNA gene was amplified using Illumina-compatible primers and the TransStart^®^ TopTaq DNA Polymerase Kit (TransGen Biotech, Beijing, China). PCR products were purified using VAHTS DNA Clean Beads (Vazyme Biotech, Nanjing, China). Library quality and concentration were assessed by Qubit quantification and agarose gel electrophoresis, and qualified libraries were sequenced on the Illumina NovaSeq 6000 platform (paired-end). Negative controls were included during DNA extraction and amplification to monitor potential contamination.

### Blood collection, flow cytometry, and ELISA analysis

After behavioral testing, mice from both groups were anesthetized *via* intraperitoneal injection of pentobarbital (30–50 mg/kg). Whole blood samples were collected, and heparin was used as an anticoagulant for flow cytometric analysis to determine the proportion of peripheral regulatory T cells (Tregs).

For serum cytokine analysis, blood samples without anticoagulant were allowed to clot and then centrifuged at 3,000 × g for 10 min at 4 °C to remove cellular debris. Serum concentrations of IL-10 (ELK Biotechnology, Cat. No. ELK1143, China), IL-6 (ELK Biotechnology, Cat. No. ELK1157, China), TNF-α (ELK Biotechnology, Cat. No. ELK1387, China), IL-1β (ELK Biotechnology, Cat. No. ELK1271, China), and lipopolysaccharide (LPS) (ELK Biotechnology, Cat. No. ELK2993, China) were measured using commercially available Enzyme-Linked Immunosorbent Assay (ELISA) kits according to the manufacturers’ instructions. Absorbance was measured using a microplate reader, and cytokine concentrations were calculated based on standard curves.

### Terminal ileum and brain tissue pathology

After blood collection from the mouse heart, mice were perfused with 4% paraformaldehyde. The terminal ileum was excised and fixed in 4% paraformaldehyde, followed by hematoxylin and eosin (HE) staining and periodic acid–Schiff (PAS) staining. Brain tissues were fixed in 4% paraformaldehyde, and hippocampal sections were prepared for HE staining, Nissl staining, and immunohistochemistry.

For immunohistochemical analysis, 8–10 IBA-1–positive microglial cells and 8–10 GFAP-positive astrocytes were randomly selected from multiple sections for each mouse. For microglia, morphological parameters including branch number, junction number, and end-point to branch ratio were quantified for each cell and averaged to obtain a single value per mouse; average optical density (AOD) of IBA-1 staining was calculated per mouse using the same approach. For astrocytes, the number of GFAP-positive cells and the AOD of GFAP staining were quantified and averaged per mouse. Group comparisons were performed using mouse-averaged values for all parameters.

For PAS analysis, well-oriented villi and crypts were randomly selected from multiple sections. For each mouse, 8–10 villi and 8–10 crypts were analyzed. The number of PAS-positive goblet cells per villus, the percentage of PAS-positive goblet cells relative to total epithelial cells per villus, as well as the crypt area and integrated density (IntDen) were quantified. All measurements were averaged to obtain a single value per mouse, and group comparisons were performed using mouse-averaged values.

### Western blot analysis

Hippocampal tissues from TD-FMT and ASD-FMT mice (*n* = 5 per group) were homogenized in RIPA lysis buffer supplemented with protease and phosphatase inhibitors. Protein concentration was quantified *via* BCA protein assay, and equal amounts of protein (30 µg per sample) were separated by 10% SDS–PAGE before transfer onto PVDF membranes. Membranes were blocked with 5% non-fat milk for 1 h at room temperature, then incubated overnight at 4 °C with primary antibodies against IBA-1 (1:2000; *Oasis, OB-PRB029*), GFAP (1:5000; *Oasis, OB-PRB005*), and GAPDH (1:5000; *Proteintech, 81640-5-RR*). Following washes, membranes were incubated with HRP-conjugated secondary antibodies (1:3000; *Affinity*) for 1 h at room temperature. Protein bands were visualized using an ECL chemiluminescence kit. Protein expression levels were normalized to the internal control GAPDH.

### Brain tissue proteomics

Consistent with previous CNS proteomics studies, we performed exploratory TMT-based proteomic profiling using *n* = 3 (Herrero-Labrador et al., 2023; Lacovich et al., 2025) biologically independent mice per group, which were randomly selected from each experimental group.After blood collection *via* heart puncture, the brains were harvested following perfusion with precooled PBS. The brain tissues were subjected to Tandem Mass Tag (TMT) quantitative proteomics analysis. This technique involves labeling peptide N-termini or lysine side-chain amino groups with isotope-labeled reagents, followed by analysis using high-resolution tandem mass spectrometry (MS/MS). It enables the simultaneous comparison of protein expression across up to 16 samples, making it a widely used high-throughput screening method in recent quantitative proteomics studies (Ross et al., 2004). The analytical workflow of this project comprises two main stages: mass spectrometry experiments and data analysis. The mass spectrometry analysis includes protein extraction, peptide digestion, TMT labeling, chromatographic fractionation, liquid chromatography-tandem mass spectrometry (LC-MS/MS) data acquisition, and database searching. Proteomic findings were subsequently validated through qPCR in the full cohort.

### Quantitative Real-Time PCR

Total RNA was extracted from intestinal tissues using RNA extraction kit (FOREGENE, RE-03011, China) according to the manufacturer’s instructions. RNA concentration and purity were determined by spectrophotometry. Complementary DNA (cDNA) was synthesized from 1 µg of total RNA using a reverse transcription kit (TransGen Biotech, AE311, China).

Quantitative real-time PCR (qRT-PCR) was performed using SYBR Green Master Mix (TransGen Biotech, AQ601, China) on a real-time PCR detection system. The amplification conditions were as follows: initial denaturation at 95 °C for 30 s, followed by 40 cycles of denaturation at 95 °C for 5 s and annealing/extension at 60 °C for 30 s. Each sample was analyzed in triplicate.

Gene expression levels were normalized to the internal control gene Gapdh, and relative mRNA expression was calculated using the 2^−^^ΔΔCt^ method, with the TD-FMT group used as the calibrator. Primer sequences used for qRT-PCR are listed in Supplement 5.

### Statistical analysis

Statistical analyses were performed using GraphPad Prism (version 10.1.2; GraphPad Software, San Diego, CA, USA). Continuous variables were first assessed for distribution characteristics using descriptive statistics. The normality of the data was evaluated by the Shapiro–Wilk test, and homogeneity of variance was assessed using Levene’s test. Based on the test results, the appropriate analysis method was selected: if the data were normally distributed with homogeneity of variance, unpaired Student’s *t*-test was used; if the data were normally distributed but with unequal variances, Welch’s *t*-test was applied; and if the data were not normally distributed, the Mann–Whitney U test was used. A *p*-value of < 0.05 was considered statistically significant. Data presentation was determined based on the distribution characteristics: normally distributed data were expressed as mean ± standard error of the mean (Mean ± SEM), while non-normally distributed data were expressed as median and interquartile range (Median, IQR).

## Results

### Behavioral alterations induced by ASD-FMT in mice

**Figure 1:**
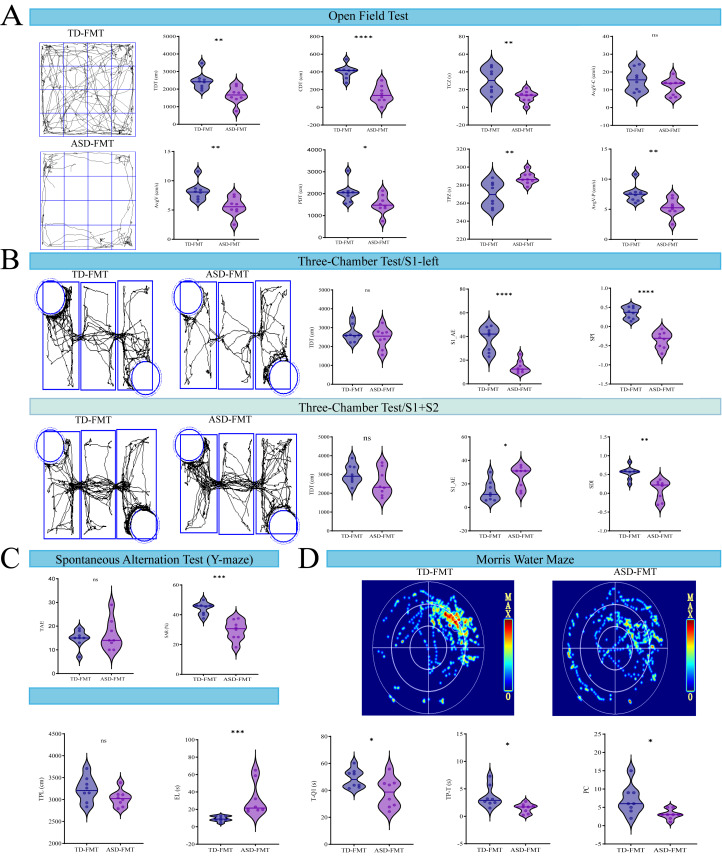
Effects of ASD-FMT on behavioral assessment in mice. (A) Representative trajectories and key behavioral metrics of mice in the open field test. (B) Representative trajectories and key behavioral metrics of mice in the three-chamber social test. (C) Comparison of Total Arm Entries and Alternation Rate (%) in the Y-maze Spontaneous Alternation Test. (D) Representative heatmap and key behavioral metrics in the Morris Water Maze (MWM).TDT: Total distance traveled (cm); AvgV: Average velocity (cm/s); CDT: Center distance traveled (cm); PDT: Peripheral distance traveled (cm); TCZ: Time in center zone (s); TPZ: Time in peripheral zone (s); AvgV-C: Average velocity in center (cm/s). S1_AE: Approaches to left cage; SPI: Social Preference Index = (S1_IT − S2_IT) / (S1_IT + S2_IT); S1_IT: Interaction time with left cage; S2_IT: Interaction time with right cage; SDI: Social Discrimination Index = (S2_IT − S1_IT) / (S1_IT + S2_IT); TAE: Total arm entries; SAR (%): Alternation rate (%); TPL: Total path length (cm); EL: Escape latency (s); T-Q1: Time in quadrant 1 (s); TP-T: Time in target platform zone (s); PC: Platform crossings. *P < 0.05, **P < 0.01, ***P < 0.001, ****P < 0.0001, ns (*P* > 0.05, not significant), compared to TD-FMT.

To assess the effects of fecal microbiota transplantation on mouse behavior, a battery of behavioral tests was performed, including the open field test, three-chamber social test, Y-maze spontaneous alternation test, and Morris water maze. In the open field test, mice in the ASD-FMT group exhibited significant differences in locomotor activity and exploratory behavior compared with the TD-FMT group. Specifically, total distance traveled (TDT) and average velocity (AvgV) were significantly altered. In addition, ASD-FMT mice showed reduced center distance traveled (CDT) and decreased time spent in the center zone (TCZ), while time spent in the peripheral zone was increased (Fig. 1A). In the three-chamber social test, differences in social interaction patterns were observed between the two groups (Fig. 1B). During the social preference phase (S1-left phase), ASD-FMT mice showed fewer approaches to the chamber containing the unfamiliar mouse (S1_AE) compared with TD-FMT mice. During the social novelty phase (S1+S2 phase), TD-FMT mice spent more time interacting with the novel unfamiliar mouse, resulting in a positive Social Discrimination Index (SDI). In contrast, ASD-FMT mice exhibited a significantly reduced SDI, reflecting altered social novelty preference. In the Y-maze spontaneous alternation test, there was no significant difference in total arm entries (TAE) between the two groups. However, the spontaneous alternation rate (SAR) was significantly lower in the ASD-FMT group compared with the TD-FMT group (Fig. 1C).

Spatial learning and memory were further evaluated using the Morris water maze (MWM). ASD-FMT mice displayed a prolonged escape latency (EL) during the training sessions. In the probe trial, ASD-FMT mice spent less time in the target quadrant (T-Q1) and showed fewer platform crossings (PC) compared with TD-FMT mice (Fig. 1D).

### 16S rRNA sequencing reveals microbiota composition and functional variations Post-FMT

#### Gut microbiota composition after FMT

**Figure 2:**
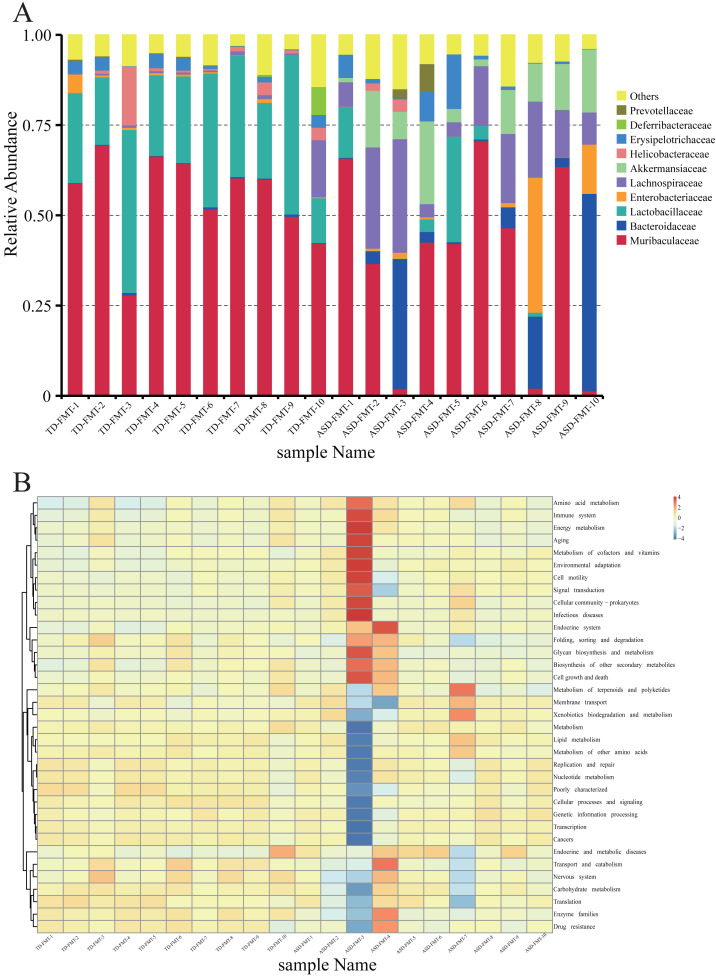
The family-level stacked bar plot. (A) shows distinct microbiota profiles between the two groups. KEGG pathway prediction based on PICRUSt analysis further revealed functional (B) differences between TD-FMT and ASD-FMT microbiota.

Family-level taxonomic composition and predicted KEGG functional pathways following FMT are shown in Fig. 2. Across all ten samples in the TD-FMT group, the bacterial community was dominated by Muribaculaceae, Bacteroidaceae, Lactobacillaceae, and Lachnospiraceae, with relatively stable inter-individual variation.

In contrast, the ASD-FMT group displayed a markedly different microbial composition, characterized by increased proportions of Enterobacteriaceae, Lachnospiraceae, Erysipelotrichaceae, and Helicobacteraceae, as well as a noticeable expansion of several low-abundance taxa.

#### Functional prediction of microbial communities

The ASD-FMT group showed higher predicted activity in pathways related to immune system processes, infectious disease, cellular stress responses, and environmental adaptation.

In contrast, the TD-FMT group exhibited greater representation of pathways associated with carbohydrate metabolism, lipid metabolism, amino acid metabolism, and genetic information processing.

### Serum biochemical profiles: inflammatory cytokines and LPS

**Figure 3:**
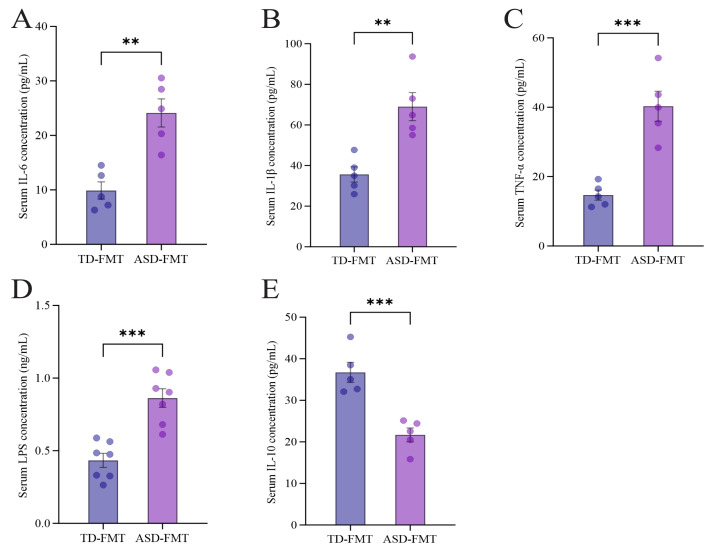
Serum inflammatory cytokine and LPS levels measured by ELISA. Serum concentrations of IL-6 (A), IL-1*β* (B), TNF-*α* (C), LPS (D), and IL-10 (E) were determined by ELISA. Data are presented as mean ± SEM. * *P* < 0.05, ** *P* < 0.01, *** *P* < 0.001 *vs.* TD-FMT.

Serum inflammatory cytokine levels and LPS concentrations were quantified by ELISA, as shown in Fig. 3. Serum ELISA analysis revealed significant alterations in inflammatory mediator levels between the ASD-FMT and TD-FMT groups (unpaired *t*-test). Specifically, the levels of pro-inflammatory cytokines (IL-6, TNF-α, IL-1β) and LPS were significantly elevated in the ASD-FMT group compared with the TD-FMT group, whereas the concentration of anti-inflammatory cytokine IL-10 was significantly reduced in the ASD-FMT group.

### Peripheral blood treg cell flow cytometry

**Figure 4:**
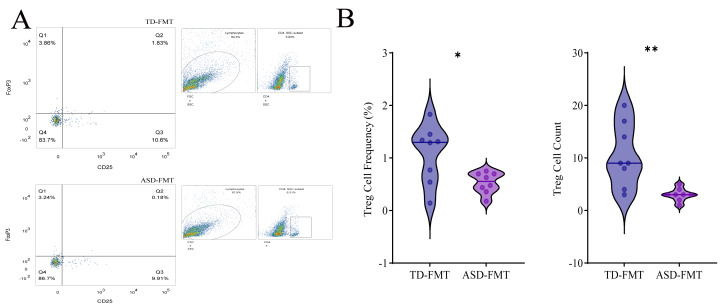
Effect of ASD-FMT on Treg expression in mice. (A) Flow cytometry gating results showing the proportion of Treg cells in the Q2 quadrant (and other relevant regions) in both groups. (B) Comparison of Treg cell count and proportion between the two groups. *P < 0.05, **P < 0.01, compared with TD-FMT.

Flow cytometry analysis revealed that the proportion and count of Treg cells in the peripheral blood of the ASD-FMT group were significantly lower than those in the TD-FMT group. According to the flow cytometry gating results, the percentage of Treg cells (CD25^+^Foxp3^+^) in quadrant Q2 of the ASD-FMT group was 0.24%, significantly lower than the 3.86% observed in the TD-FMT group. Additionally, both the Treg cell count and overall proportion were markedly reduced in the ASD-FMT group (Figs. 4A, 4B).

### HE and PAS staining of the ileum

**Figure 5:**
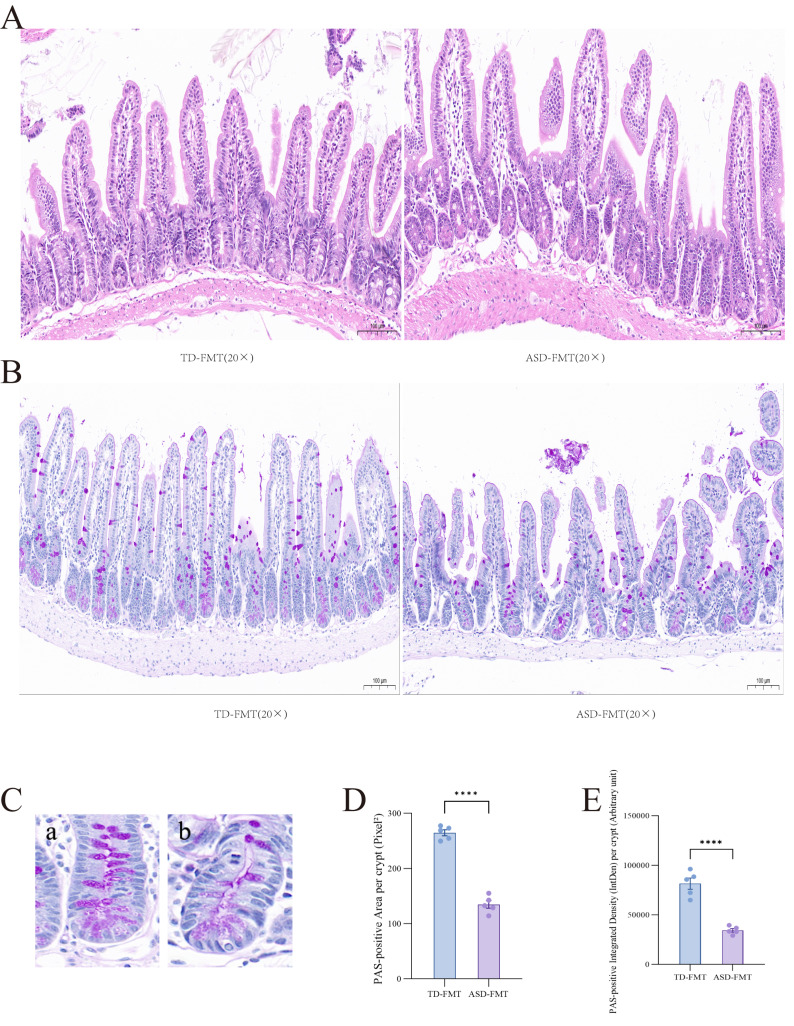
HE and PAS staining of the terminal ileum in both groups of mice. (A) HE staining of the terminal ileum (20 ×, scale bar = 100 μm). (B) PAS staining of the terminal ileum (20 ×, scale bar = 100 μm). (C) Representative images of individual intestinal crypts from TD-FMT (a) and ASD-FMT (b) mice used for quantitative analysis. (D) Quantification of crypt area. (E) Quantification of integrated density (IntDen) of PAS staining in intestinal crypts. ****P < 0.0001.

Histological features of the terminal ileum assessed by HE and PAS staining are shown in Fig. 5. Histological examination of the terminal ileum was performed using HE and PAS staining. Under light microscopy at 20 × magnification, both the TD-FMT and ASD-FMT groups preserved the overall intestinal architecture, including intact mucosa and villus–crypt organization, with no evidence of overt pathological damage, such as extensive villus loss or glandular necrosis.

In HE-stained sections (Fig. 5A), although the general morphology was comparable between groups, the ASD-FMT group exhibited less regular villus alignment, with occasional irregularly shaped villi and uneven inter-villus spacing. In addition, the boundary between the mucosal and submucosal layers appeared less clearly defined in the ASD-FMT group, suggesting subtle structural alterations.

PAS staining was used to evaluate goblet cells and crypt morphology (Fig. 5B). Compared with the TD-FMT group, the ASD-FMT group showed a reduced density of PAS-positive goblet cells and weaker staining intensity, indicating impaired mucin-producing capacity. Consistent with these observations, quantitative analysis revealed that both crypt area and integrated density (IntDen) were significantly higher in the TD-FMT group than in the ASD-FMT group, consistent with altered crypt morphology and PAS staining intensity.

### HE and Nissl staining of the hippocampus

**Figure 6:**
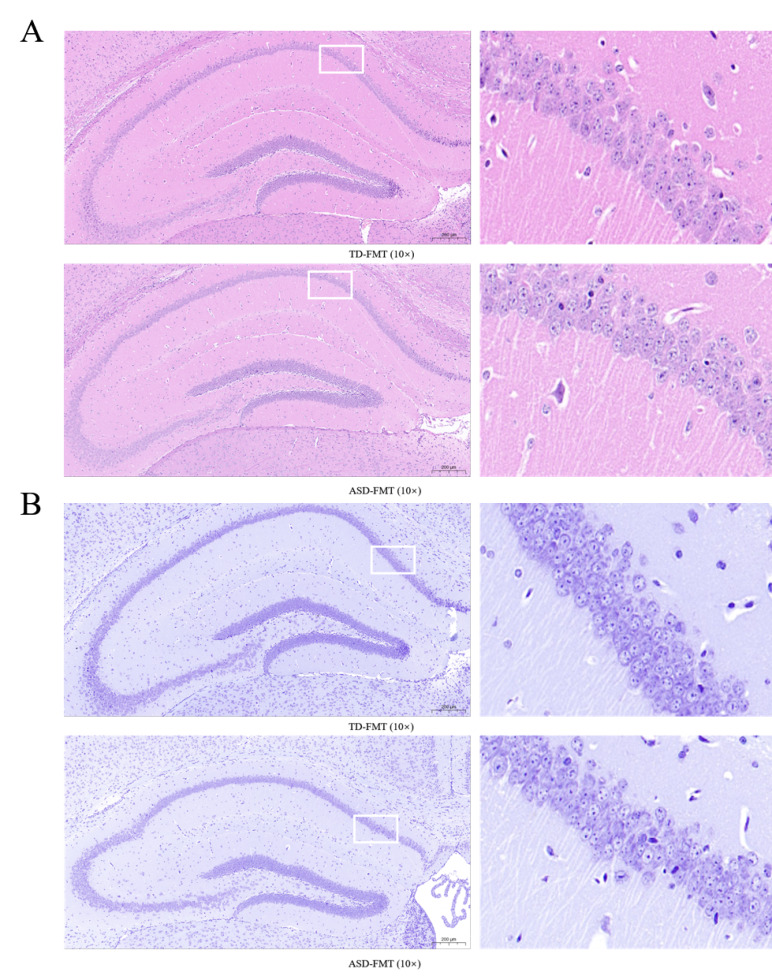
HE and Nissl staining of the hippocampus in both groups of mice. (A) HE staining of the hippocampus (10 ×, scale bar = 200 μm). (B) Nissl staining of the hippocampus (10 ×, scale bar = 200 μm).

HE and Nissl staining of the hippocampus showed preserved laminar organization in both groups. Compared with TD-FMT mice, the ASD-FMT group exhibited subtle alterations in neuronal arrangement, characterized by mildly reduced cellular compactness and decreased Nissl staining intensity in selected hippocampal regions (Fig. 6).

### Immunohistochemistry and cell skeleton analysis

**Figure 7:**
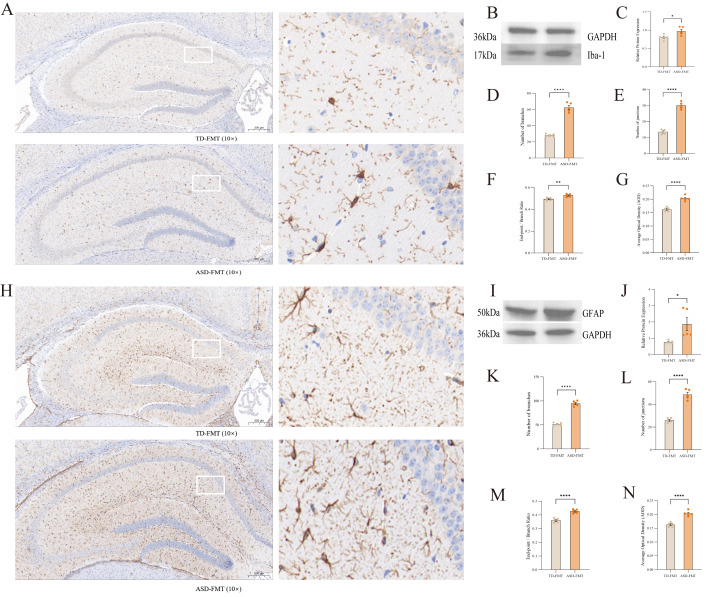
IBA-1 and GFAP immunohistochemistry, Western blot, and cell skeleton analysis in ASD-FMT and TD-FMT mice. (A) Representative IBA-1 immunostaining in the hippocampus of TD-FMT and ASD-FMT mice. Scale bar = 50 µm. (B) Western blot of IBA-1 protein expression. (C) Quantification of IBA-1 protein levels from Western blot (*n* = 5 per group). (D–G) Quantitative analysis of IBA-1 microglial morphology: (D) Branches, (E) Junctions, (F) Average Optical Density (AOD), and (G) End-point / Branch Ratio. (H) Representative GFAP immunostaining in the hippocampus of TD-FMT and ASD-FMT mice. Scale bar = 50 µm. (I) Western blot of GFAP protein expression. (J) Quantification of GFAP protein levels from Western blot (*n* = 5 per group). (K–N) Quantitative analysis of GFAP astrocyte morphology: (K) Branches, (L) Junctions, (M) AOD, and (N) End-point/Branch Ratio. Data are presented as mean ± SEM. * *P* < 0.05, ** *P* < 0.01, *** *P* < 0.001, **** *P* < 0.0001 *versus* TD-FMT group (statistical analysis as described in Methods). Target proteins and GAPDH were detected on the same membrane. Full-length, uncropped blots are provided in the Supplementary Materials.

Microglial and astrocytic alterations in the hippocampus assessed by immunohistochemistry, Western blotting, and morphological analysis are summarized in Fig. 7. To evaluate the effect of fecal microbiota transplantation (FMT) on hippocampal glial morphology, we quantitatively analyzed morphological parameters, including the number of branches, number of junctions, end-point/branch ratio, and average optical density (AOD), by immunostaining for ionized calcium-binding adapter molecule 1 (IBA-1, a microglial marker) and glial fibrillary acidic protein (GFAP, an astrocytic marker). Compared with the typically developing (TD)-FMT group, mice in the autism spectrum disorder (ASD)-FMT group exhibited significantly higher values for all four morphological indices in both IBA-1-positive microglia and GFAP-positive astrocytes in the hippocampus (all *P* < 0.05).

### Brain proteomic profiling reveals enrichment of neurodevelopment-related pathways

**Figure 8:**
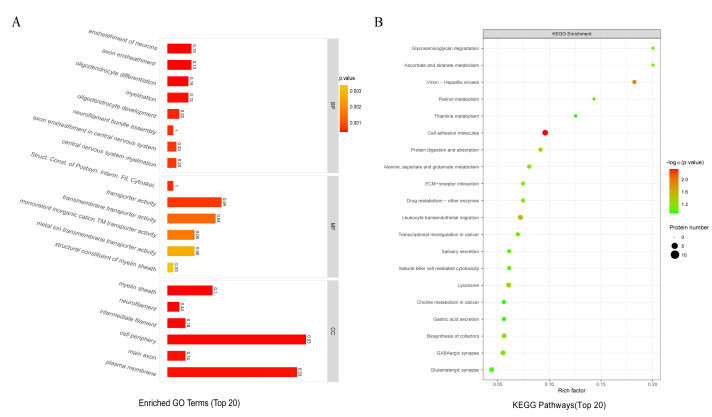
LC-MS/MS detection of brain tissue followed by GO and KEGG enrichment analysis. LC-MS/MS detection of brain tissue followed by GO and KEGG enrichment analysis. (A) GO enrichment analysis; (B) KEGG enrichment analysis.

The GO enrichment analysis of TMT-based brain tissue proteomics revealed that differentially expressed proteins (DEPs) were enriched in biological processes related to neuron ensheathment, axon ensheathment, oligodendrocyte differentiation, and myelination. In addition, enrichment was observed in molecular function categories associated with the structural organization of postsynaptic intermediate filament cytoskeletons and myelin sheath components (Fig. 8A).

KEGG pathway enrichment analysis further indicated that these DEPs were enriched in pathways related to glycosaminoglycan degradation and ascorbate and aldarate metabolism (Fig. 8B).

### Quantitative Real-Time PCR

Quantitative real-time PCR analysis further demonstrated that the transcriptional changes observed in brain tissues were consistent with the proteomic findings. Moreover, the expression of inflammatory- and barrier-related genes in both brain and intestinal tissues showed significant differences between the ASD-FMT and TD-FMT groups (Supplement 6).

## Discussion

In this study, we examined the effects of FMT from children with ASD on behavioral performance, gut microbial composition, peripheral immune parameters, and histological features in antibiotic-pretreated mice. Our findings describe associations between ASD-associated gut microbiota and a range of host behavioral, immunological, and tissue-level alterations, highlighting potential links between microbial composition and host responses relevant to ASD (Alharthi et al., 2022).

### Behavioral alterations following transplantation of ASD-associated microbiota

Mice receiving fecal microbiota from children with ASD exhibited altered behavioral performance across multiple paradigms (Prince et al., 2024), including open field exploration, social interaction, and spatial learning and memory tasks (Hirota & King, 2023; Yan & Rein, 2022). Collectively, these paradigms probe core functional domains such as locomotor activity, anxiety-related behavior, sociability, and cognitive processing, which are commonly evaluated in experimental models investigating neurodevelopmental disorders (Malkova et al., 2012).

Consistent with previous FMT-based studies, ASD-FMT mice showed reduced social preference, impaired discrimination of social novelty (Banker et al., 2021), and deficits in spatial working memory and learning performance. These behavioral changes are compatible with altered neural processing and circuit function reported in ASD-related research and suggest that ASD-associated gut microbiota may influence host behavioral phenotypes through complex host–microbiota interactions (Zohny et al., 2023).

### Gut microbial composition changes following FMT

16S rRNA gene sequencing demonstrated that fecal microbiota transplantation resulted in clearly distinct gut microbial profiles between the ASD-FMT and TD-FMT groups. In the TD-FMT group, the bacterial community was consistently dominated by *Muribaculaceae*, *Bacteroidaceae, Lactobacillaceae*, and *Lachnospiraceae,* with relatively low inter-individual variability, indicating a stable and balanced microbial structure following transplantation.

In contrast, mice receiving ASD-derived microbiota exhibited a markedly altered community composition, characterized by increased relative abundances of *Enterobacteriaceae*, *Erysipelotrichaceae*, *Helicobacteraceae*, and specific members of *Lachnospiraceae*, along with an expansion of several low-abundance taxa. Notably, many of these taxa have been repeatedly reported to be enriched or dysregulated in clinical ASD cohorts and experimental ASD-related models, supporting the biological and translational relevance of the donor-derived microbial signatures observed in this study (Chang et al., 2025; Peralta-Marzal et al., 2024).

These ASD-associated microbial profiles co-occurred with alterations in behavioral performance, peripheral immune parameters, and tissue-level phenotypes, suggesting that shifts in gut microbial community structure may be linked to host functional changes across multiple biological domains. While the present study does not dissect the individual contributions of specific microbial taxa or metabolites, the observed community-level alterations provide a meaningful ecological context in which ASD-associated microbiota may influence host physiology.

### Peripheral immune alterations following ASD-FMT

Peripheral immune profiling revealed marked alterations in circulating immune parameters following ASD-derived fecal microbiota transplantation. Flow cytometry analysis demonstrated a significant reduction in both the proportion and absolute number of peripheral regulatory T cells (Treg) in ASD-FMT mice compared with TD-FMT controls. In parallel, serum ELISA analysis showed significantly elevated levels of pro-inflammatory mediators, including IL-6, TNF-α, IL-1β, and lipopolysaccharide (LPS), along with a concomitant reduction in the anti-inflammatory cytokine IL-10.

Regulatory T cells are central regulators of immune homeostasis, and reduced Treg abundance together with a pro-inflammatory cytokine profile has been repeatedly reported in subsets of individuals with ASD (Palanivelu et al., 2024; Pellegrini et al., 2020; Zhou et al., 2025). The immune alterations observed in ASD-FMT mice therefore recapitulate key features of immune imbalance described in clinical ASD studies, supporting the biological relevance of the donor-derived microbiota (Beopoulos et al., 2021; Lukens & Eyo, 2022; Petropoulos et al., 2025).

Taken together, these findings indicate that ASD-associated gut microbiota are linked to a shift in peripheral immune equilibrium toward a pro-inflammatory milieu. While the present study focused on circulating Treg cells and selected cytokines, the consistency of these immune changes with behavioral and histological alterations suggests that peripheral immune modulation represents an important host response accompanying ASD-associated microbial colonization (Cao et al., 2021; Zhao et al., 2021).

### Intestinal and hippocampal histological alterations following ASD-FMT

Histological examination of the terminal ileum revealed structural differences between groups. ASD-FMT mice exhibited disrupted villus organization and a significantly reduced abundance of goblet cells abundance compared with TD-FMT controls. Goblet cells are essential for maintaining the intestinal mucus layer and epithelial barrier function, and decreased goblet cell density has been widely associated with altered gut barrier properties in conditions involving microbiota dysbiosis. The intestinal structural changes observed in ASD-FMT mice are consistent with reports from both clinical ASD cohorts and experimental models (Mathew et al., 2024), supporting the sensitivity of gut tissue architecture to donor-derived microbial composition.

HE and Nissl staining demonstrated that the overall hippocampal laminar structure was preserved in both groups. Compared with TD-FMT mice, the ASD-FMT group showed mildly altered neuronal arrangement and reduced Nissl staining intensity in selected hippocampal regions, indicating subtle changes in neuronal morphology rather than overt neuronal loss. In parallel, immunohistochemical and Western blot analyses demonstrated significantly increased expression of the glial activation markers IBA-1 and GFAP in the ASD-FMT group compared with TD-FMT mice. Elevated IBA-1 and GFAP expression reflects altered microglial and astrocytic status, which has been repeatedly reported in association with ASD-related neuroimmune alterations and microbiota-associated brain changes (Rexrode et al., 2024).

Together, these intestinal and hippocampal histological findings indicate that ASD-associated microbiota colonization is accompanied by coordinated structural changes in both peripheral and central tissues. The convergence of epithelial alterations in the gut and glial-associated changes in the hippocampus provides histological evidence consistent with a link between donor-derived microbial profiles and host tissue responses relevant to ASD-associated phenotypes.

### Exploratory brain proteomics and pathway associations

Exploratory TMT-based proteomic profiling revealed differential protein expression patterns in ASD-FMT mice that were enriched in neurodevelopment-related biological processes, including neuronal ensheathment, oligodendrocyte differentiation, and myelination, as well as metabolic pathways such as glycosaminoglycan degradation and ascorbate metabolism. These pathways are critically involved in neural circuit maturation, axonal integrity, and oxidative homeostasis, and have been repeatedly implicated in neurodevelopmental and neurological conditions (She et al., 2026; Yousefi et al., 2022), including ASD (Won & Maher, 2026).

Notably, several of the identified pathways converge on processes relevant to white matter organization and cellular metabolic regulation, providing a molecular context that aligns with the observed behavioral alterations and histological changes in the hippocampus (Hanson et al., 2025). Targeted qRT-PCR validation of selected genes showed expression patterns consistent with the proteomic trends, lending additional support to the robustness of the pathway-level findings. Together, these data suggest that ASD-associated microbiota colonization is accompanied by coordinated alterations in brain molecular networks linked to neurodevelopmental regulation.

In addition, the relatively small sample size, particularly for the exploratory proteomic analysis, should be considered when interpreting the present findings, and future studies with larger cohorts will be necessary to further validate these observations.

### Limitations and future directions

This study has several limitations. First, while 16S rRNA sequencing confirmed microbial composition differences following FMT, functional microbial outputs such as metabolite profiles were not assessed. Second, immune analysis was limited to Treg frequency and selected cytokines, restricting conclusions regarding immune mechanisms. Third, behavioral and histological alterations were analyzed at a single time point, precluding conclusions about temporal dynamics.

Future studies integrating microbial metabolomics, expanded immune profiling, and longitudinal behavioral assessments will be critical to clarify how ASD-associated microbiota influences host physiology and behavior. Targeted manipulation of specific microbial taxa may further help to disentangle associative from causal relationships.

## Conclusions

In summary, fecal microbiota transplantation from children with ASD was associated with altered gut microbial composition, changes in peripheral immune parameters, intestinal and hippocampal histological alterations, and behavioral differences in recipient mice. These findings support the concept that ASD-associated gut microbiota is linked to host behavioral and biological outcomes, providing a multidimensional framework for future studies investigating microbiota–host interactions relevant to neurodevelopmental disorders.

## Supplemental Information

10.7717/peerj.20951/supp-1 Supplemental Information 1 Inclusion and Exclusion Criteria for Donors

10.7717/peerj.20951/supp-2 Supplemental Information 2 FMT Donor Information

10.7717/peerj.20951/supp-3 Supplemental Information 3 Changes in gut microbial DNA after antibiotic treatment and body weight during transplantation

10.7717/peerj.20951/supp-4 Supplemental Information 4 Data of behavioral testing

10.7717/peerj.20951/supp-5 Supplemental Information 5 Primer sequences used for quantitative real-time PCR

10.7717/peerj.20951/supp-6 Supplemental Information 6 Quantitative real-time PCR results of relative mRNA expression of target genes in hippocampal and intestinal tissues

10.7717/peerj.20951/supp-7 Supplemental Information 7 Full-length, uncropped Western blots

10.7717/peerj.20951/supp-8 Supplemental Information 8 The ARRIVE guidelines

## Additional Information and Declarations

### Data Availability

The following information was supplied regarding data availability: The 16S rRNA sequencing data generated in this study is available at Zenodo: Gao, Y. (2025). 16S rRNA Sequencing Analysis of Intestinal Microbiota Structure in Mice After Fecal Microbiota Transplantation [Data set]. Zenodo. https://doi.org/10.5281/zenodo.17954992. The TMT-based proteomics data generated from mouse brain tissues in this study is available at iProx: IPX0013294000.
