# Supplementary material for: Behavioral and biological alterations following transplantation of ASD-associated gut microbiota in mice
Source: PeerJ. 2026 Mar 24;14:e20951. doi: 10.7717/peerj.20951 (PMC13024247; doi:10.7717/peerj.20951)
Supplement: Supplemental Information 1 [file peerj-14-20951-s001.docx]

Supplement 1

**Inclusion and Exclusion Criteria for Donors in the Autism Spectrum Disorder (ASD) Group:**

**Inclusion Criteria:**

**Confirmed Diagnosis:**

Diagnosis of Autism Spectrum Disorder (ASD) according to the *Diagnostic and Statistical Manual of Mental Disorders, Fifth Edition* (DSM-5) criteria.

Diagnosis confirmed by two pediatric psychiatrists or specialists in developmental and behavioral pediatrics.

**Age Range:** Male children aged 3 to 5 years (preferably 4 to 5 years).

**Symptom Severity:**

Childhood Autism Rating Scale (CARS) total score ≥ 32 (moderate to severe symptoms).

**Co-occurring Gastrointestinal Symptoms:**

At least one gastrointestinal symptom associated with ASD, such as chronic constipation (≥3 times/week), diarrhea (Bristol stool scale ≥6), or abdominal bloating/pain.

**No Interfering Treatments:**

No use of antibiotics, probiotics, immunosuppressants, or neuroactive medications (e.g., risperidone) within the past 3 months.

No involvement in restrictive dietary interventions (e.g., ketogenic diet, gluten-free diet).

**Exclusion Criteria:**

**Co-morbidities:**

Congenital intestinal disorders (e.g., inflammatory bowel disease, celiac disease), genetic syndromes (e.g., Fragile X syndrome, tuberous sclerosis), epilepsy, or severe brain injury.

Acute infections (fever >38°C or diarrhea >3 times/day within the past 72 hours).

**Previous Interventions:**

Prior fecal microbiota transplantation (FMT) or intestinal surgery.

Long-term use (>1 month) of proton pump inhibitors (e.g., omeprazole).

**Sample Quality Issues:**

Visible mucus, blood, or undigested food residues in fecal samples (fecal occult blood and parasitic screening required).

**Inclusion and Exclusion Criteria for Healthy Control Group:**

**Inclusion Criteria:**

**Health Status Confirmation:**

Normal results from routine pediatric health checkups (growth curve within the WHO P10 to P90 range).

No history of neurodevelopmental disorders or psychiatric conditions, confirmed by a pediatrician.

**Age and Gender Matching:**

Strictly matched with ASD donor group: male children aged 3 to 5 years (±3 months).

Reside in the same geographical area (to control for dietary and environmental exposure differences).

**Developmental and Behavioral Screening:**

CARS total score < 25 (no ASD traits).

Age and Developmental Questionnaire (ASQ-3) scores in all five domains (communication, gross motor, fine motor, problem-solving, personal-social) ≥ threshold.

**Normal Gastrointestinal Function:**

No chronic abdominal pain, diarrhea (Bristol scale types 4-5), or constipation (≥4 bowel movements/week).

Fecal occult blood, parasite, and pathogen screening within normal limits.

**Exclusion Criteria:**

Exclusion if any of the following conditions are met:

**Disease-related Exclusion:**

Diagnosed with allergic diseases (e.g., asthma, atopic dermatitis), autoimmune diseases, or metabolic disorders in the past 6 months.

History of prematurity (<37 weeks gestation) or birth asphyxia (Apgar score ≤7).

**Intervention and Medication Exclusion:**

Use of antibiotics, probiotics, immunomodulators, or gastrointestinal motility drugs (e.g., lactulose) in the past 3 months.

Special diets (e.g., hydrolyzed protein formula, gluten-free diet).

**Family History Exclusion:**

First-degree relatives with a history of ASD, schizophrenia, or inflammatory bowel disease.

**Demographic Characteristics of the Two Groups of Children:**

**Statistical Analysis:**
All continuous variables were tested for normality using the Shapiro-Wilk test (P > 0.05). Group comparisons were performed using independent (unpaired) t-tests. Statistical significance was defined as two-tailed P < 0.05（Table 1）. Statistical analyses were performed using GraphPad Prism 10.1.2 software.

**Table 1: Demographic and Clinical Characteristics of Fecal Microbiota Transplantation Donors**

|  | **TD (n=5)** | **ASD (n=5)** | **p-value** |
| --- | --- | --- | --- |
| Gender (M) | 5 | 5 | 1.000 |
| Age (months) | 47.60 ± 4.59 | 47.80 ± 4.40 | 0.799 |
| Weight (kg) | 15.80 ± 0.61 | 15.78 ± 0.65 | 0.799 |
| **Perinatal Background Factors** |  |  |  |
| Mode of delivery (C-section / vaginal) | 1/4 | 2/3 | – |
| Prenatal or early postnatal antibiotic exposure (yes / no) | 1/4 | 2/3 | – |
| Maternal Age (years) | 29.00 ± 1.67 | 28.80 ± 2.42 | 0.815 |
| Gestational Age (weeks) | 38.80 ± 0.37 | 38.60 ± 0.51 | 0.749 |
| Birth Weight (g) | 3410 ± 111.10 | 3370 ± 139.3 | 0.456 |
| **Scale Scores** |  |  |  |
| CARS | 16.20 ± 1.07 | 36.00 ± 0.32 | <0.001 |

Note: Due to the small sample size, no statistical comparison was performed for categorical perinatal background variables.
