# Supplementary material for: Behavioral and biological alterations following transplantation of ASD-associated gut microbiota in mice
Source: PeerJ. 2026 Mar 24;14:e20951. doi: 10.7717/peerj.20951 (PMC13024247; doi:10.7717/peerj.20951)
Supplement: Supplemental Information 3 [file peerj-14-20951-s003.docx]

Supplement3

**1. Comparison of Fecal DNA Between Antibiotic-Treated Mice and Normal Mice After 5 Days of Antibiotic Administration**

Fecal DNA was extracted from mice using a fecal genomic DNA extraction kit (Beijing Solarbio Science & Technology Co., Ltd, Catalog No. D2700) following the method described by Guo et al. (Guo et al. 2020). The DNA concentrations of the antibiotic-treated mice and untreated control mice were compared (Figure 1).

**Figure 1.** Comparison of Fecal DNA Between Antibiotic-Treated Mice and Normal Mice After 5 Days of Antibiotic Administration


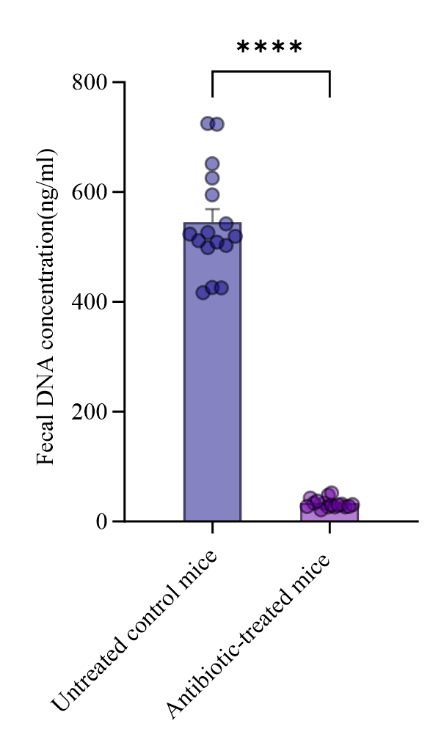


**2. Daily Weight Changes of Mice in the ASD-FMT and TD-FMT Groups During Fecal Microbiota Transplantation (FMT)**

The body weight of mice in the ASD-FMT and TD-FMT groups was measured daily during the FMT process. As shown in Figure 2, Day 1 represents the weight of the mice before FMT, with no statistically significant difference between the two groups. Days 2 to 8 correspond to the period of fecal microbiota transplantation (from Day 1 to Day 7), and Days 9 to 15 correspond to the period from the completion of FMT until the behavioral testing. On Days 1 and 2, there were no significant differences in body weight between the two groups. However, from Days 3 to 15, the body weight of the ASD-FMT group was significantly lower than that of the TD-FMT group.

**Figure 2.** Daily Body Weight Changes in ASD-FMT and TD-FMT Mice During Fecal Microbiota Transplantation


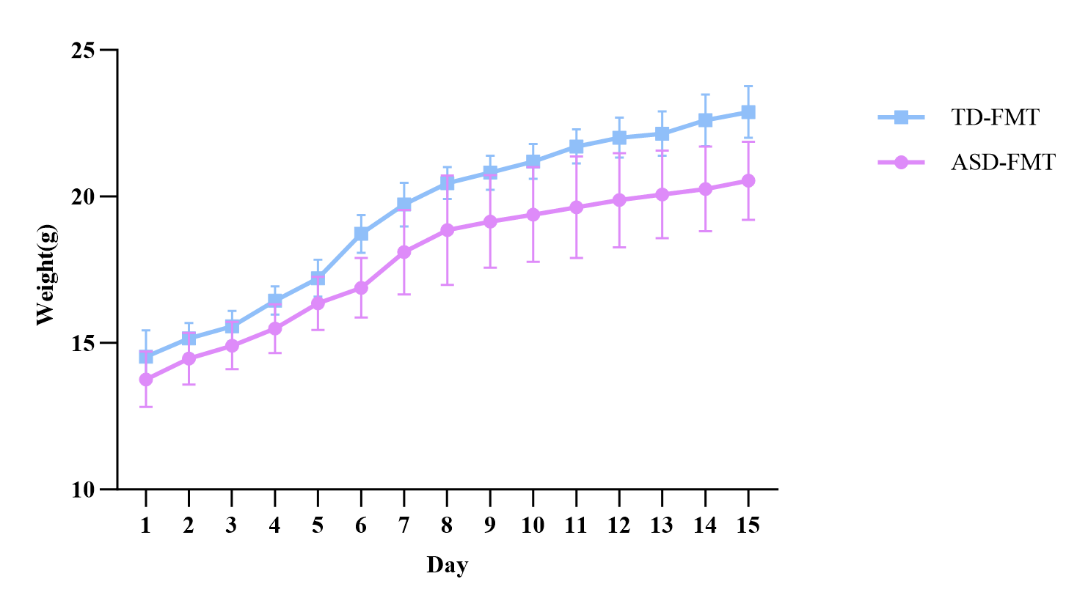


**References:**

Guo YP, Shao L, Chen MY, Qiao RF, Zhang W, Yuan JB, and Huang WH. 2020. In Vivo Metabolic Profiles of *Panax notoginseng* Saponins Mediated by Gut Microbiota in Rats. *J Agric Food Chem* 68:6835-6844. 10.1021/acs.jafc.0c01857
