# Supplementary material for: Behavioral and biological alterations following transplantation of ASD-associated gut microbiota in mice
Source: PeerJ. 2026 Mar 24;14:e20951. doi: 10.7717/peerj.20951 (PMC13024247; doi:10.7717/peerj.20951)
Supplement: Supplemental Information 5 [file peerj-14-20951-s005.docx]

Supplementary 5 Primer sequences used for quantitative real-time PCR.

Table 1. Primer sequences used for quantitative real-time PCR

| **Protein** | **Gene** | **Primer sequence** |  |  |  |
| --- | --- | --- | --- | --- | --- |
| TLR2 | *Tlr2* | F: gcgacatcaatcacctgact |  |  |  |
|  |  | R: gctgacttcatctacgggca |  |  |  |
| TLR4 | *Tlr4* | F: tggtttacacgtccatgggt |  |  |  |
|  |  | R: gcagaaacattcgccaagca |  |  |  |
| IL-1β | *Il1b* | F: gtggcagctaccgtggtctt |  |  |  |
|  |  | R: ggagcctgtggtgcagttgt |  |  |  |
| NF-κB p50 | *Nfkb1* | F: actggaagcacggatgacag |  |  |  |
|  |  | R: cctggcggatgatctccttc |  |  |  |
| iNOS | *Nos2* | F: ctatggccgctttgatgtgc |  |  |  |
|  |  | R: ttgggatgctccatggtcac |  |  |  |
| ARG1 | *Arg1* | F: acattggcttgcgagacgta |  |  |  |
|  |  | R: atcaccttgccaatccccag |  |  |  |
| CD68 | *Cd68* | F: cagggaggttgtgacggtac |  |  |  |
|  |  | R: actcggcgtctgatgtaggt |  |  |  |
| C1QA | *C1qa* | F: ctggcatccggactggtatc |  |  |  |
|  |  | R: ctttcacgcccttcagtcct |  |  |  |
| OCLN | *Ocln* | F: atcctgtctatgctcattgtga |  |  |  |
|  |  | R: tcgggttttcacagcaaaaaaga |  |  |  |
| ZO-1 | *Tjp1* | F: tcctgagccggtgtctgata |  |  |  |
|  |  | R: cctgcttgcactcctatccc |  |  |  |
| CLDN11 | *Cldn11* | F: atccccacctgccgaaaaat |  |  |  |
|  |  | R: ggggagaactgtcaacagca |  |  |  |
| BDNF | *Bdnf* | F: tacctggatgccgcaaacat |  |  |  |
|  |  | R: ccagacatgtccactgcagt |  |  |  |
| GLUA1 | *Gria1* | F: agaagaactggcaggtgacg |  |  |  |
|  |  | R: acccgatgccgttcttttct |  |  |  |
| GABRA1 | *Gabra1* | F: ggttgaccgtgagagctgaa |  |  |  |
|  |  | R: ctacaaccactgaacgggct |  |  |  |
| PLP1 | *Plp1* | F: gctttccctggcaaggtttg |  |  |  |
|  |  | R: agggaaaacagtgtggccg |  |  |  |
| MBP | *Mbp* | F: tcaggcagatctttggcgac |  |  |  |
|  |  | R: ccggctctgcttccatacaa |  |  |  |
| CNP | *Cnp* | F: cctggagaagtaccacgacg |  |  |  |
|  |  | R: gtctagacgcttgtacgcct |  |  |  |
| MOG | *Mog* | F: ccaagatgcagagcaagcac |  |  |  |
|  |  | R: tgcgatgagagtcagcacac |  |  |  |
| GJB1 | *Gjb1* | F: atctgctctaccccggctat |  |  |  |
|  |  | R: ggagggtgcgcgagcataaaga |  |  |  |
| CCNT2 | *Ccnt2* | F: cacggttaccctagagctgc |  |  |  |
|  |  | R: gttggcaggtacaccagtga |  |  |  |
| SIRT2 | *Sirt2* | F: ctgggaggtggcatggattt |  |  |  |
|  |  | R: aggcagccttgatcacagtc |  |  |  |
| CNIH2 | *Cnih2* | F: tgtcatctggcacatcatagcc |  |  |  |
|  |  | R: acatcagacagaagaggccg |  |  |  |
| CPNE6 | *Cpne6* | F: ccaatggagaccagagtgacc |  |  |  |
|  |  | R: aactcgccgatgaagtcgtg |  |  |  |
| HPCAL1 | *Hpcal1* | F: ctctgagtgtgacctctcgg |  |  |  |
|  |  | R: aggcatcttcatcacggagg |  |  |  |
| HOPX | *Hopx* | F: ggtctcacggaggagcagac |  |  |  |
|  |  | R: agtccgtaacagatctgcattcc |  |  |  |
| CPNE7 | *Cpne7* | F: gttggcattcggcaggaatg |  |  |  |
|  |  | R: cccgtcgtcattaacccgat |  |  |  |
| TMEFF2 | *Tmeff2* | F: agtgactactagctgctgtg |  |  |  |
|  |  | R: ctccttctgactggtctcgc |  |  |  |
| CRYM | *Crym* | F: atcaggcatcggtgcttctc |  |  |  |
|  |  | R: gcttcaacagcttgtggca |  |  |  |
| MUC2 | *Muc2* | F: tcctgaccaagagcgaacac |  |  |  |
|  |  | R: acagcacgacagtcttcagg |  |  |  |
| TPH1 | *Tph1* | F: cgacatcagccgagaacagt |  |  |  |
|  |  | R: gtcttccttcgcagtgagct |  |  |  |
| GAPDH | *Gapdh* | F: tgatgggtgtgaaccacgag |  |  |  |
|  |  | R: agtgatgggatggactgtgg |  |  |  |
