# Supplementary material for: Behavioral and biological alterations following transplantation of ASD-associated gut microbiota in mice
Source: PeerJ. 2026 Mar 24;14:e20951. doi: 10.7717/peerj.20951 (PMC13024247; doi:10.7717/peerj.20951)
Supplement: Supplemental Information 6 [file peerj-14-20951-s006.docx]

Supplement 6

Quantitative real-time PCR results of relative mRNA expression of target genes in hippocampal and intestinal tissues

**
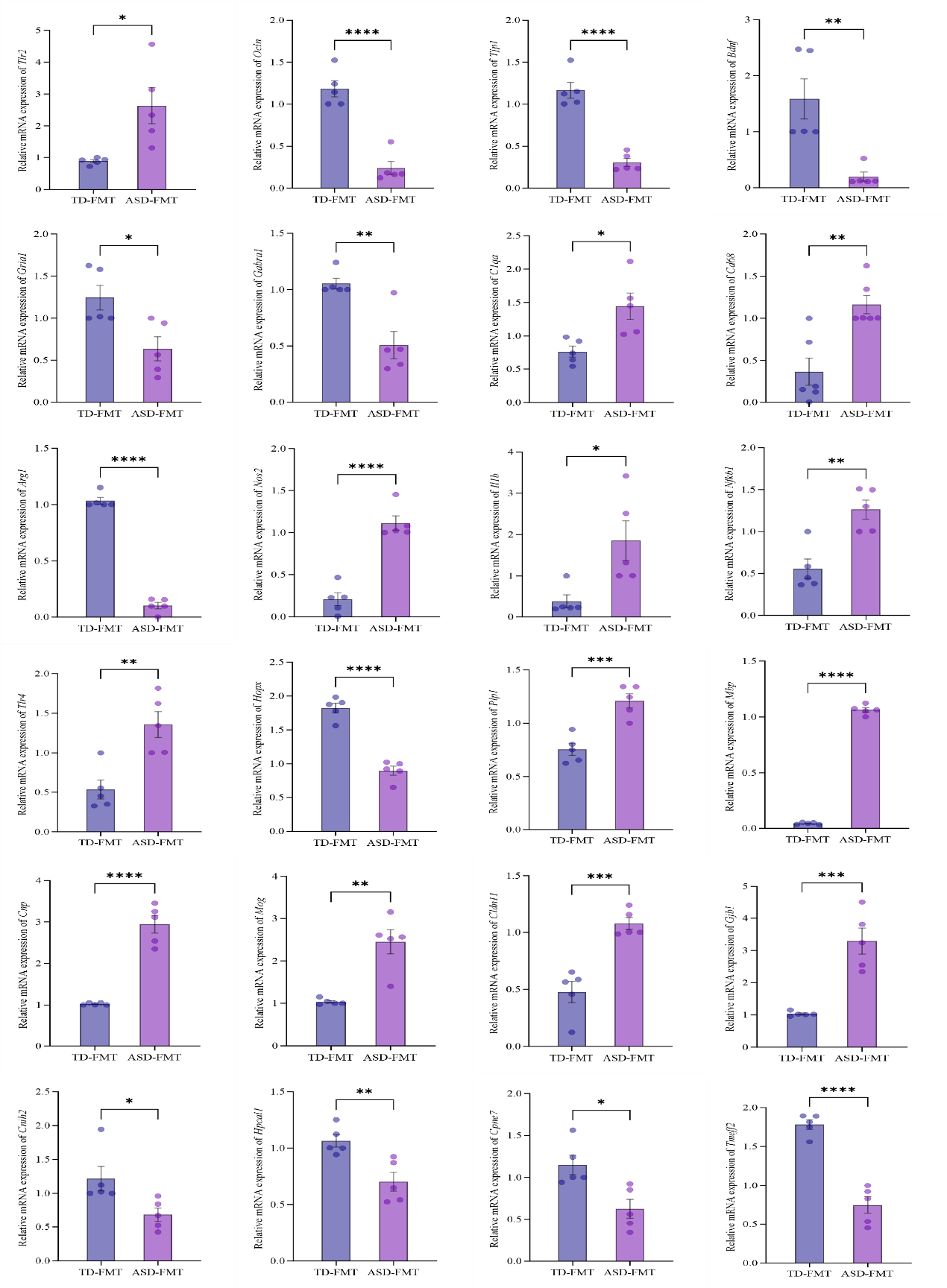
**

**Figure 1. Relative mRNA expression of genes in hippocampal tissues.**

Relative mRNA expression of target genes was determined by quantitative real-time PCR and calculated using the 2⁻^ΔΔCt^ method, with the TD-FMT group as the control.


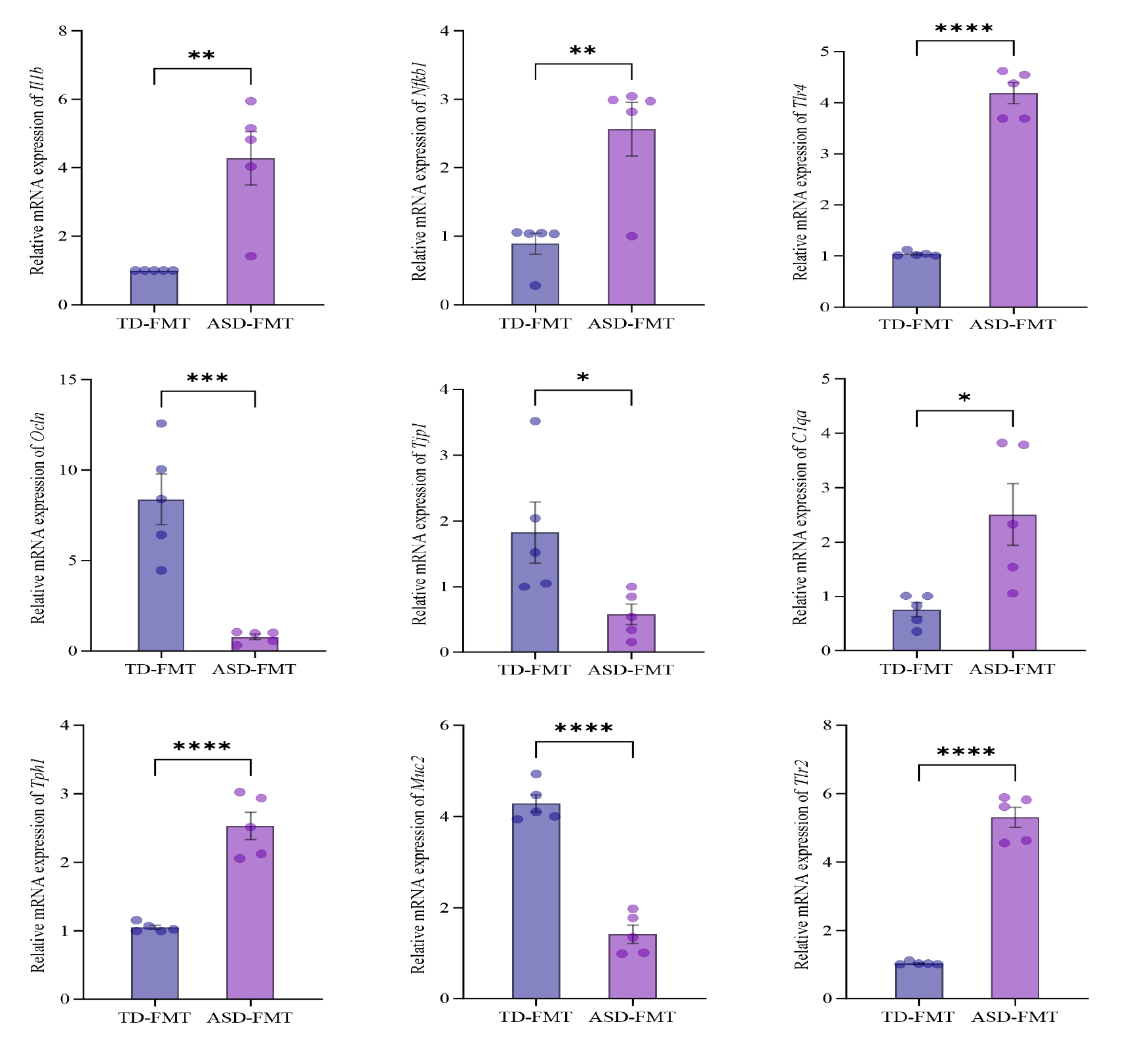


**Figure 2. Relative mRNA expression of inflammatory-related genes in intestinal tissues.**

Relative mRNA expression of target genes was determined by quantitative real-time PCR and calculated using the 2⁻^ΔΔCt^ method, with the TD-FMT group as the control.
