# Supplementary material for: Behavioral and biological alterations following transplantation of ASD-associated gut microbiota in mice
Source: PeerJ. 2026 Mar 24;14:e20951. doi: 10.7717/peerj.20951 (PMC13024247; doi:10.7717/peerj.20951)

Full-length, uncropped Western blot images.

Full-length Western blot membranes showing IBA-1 or GFAP together with the loading control GAPDH, detected on the same membrane using hippocampal protein lysates from TD-FMT and ASD-FMT mice. These images correspond to the Western blot data quantified in Figure 7 of the main manuscript. The bands used for densitometric quantification in Figure 7 were derived from these full-length membranes.

(A) IBA-1

Full-length Western blot membrane showing IBA-1 and GAPDH expression in hippocampal samples from TD-FMT and ASD-FMT mice.

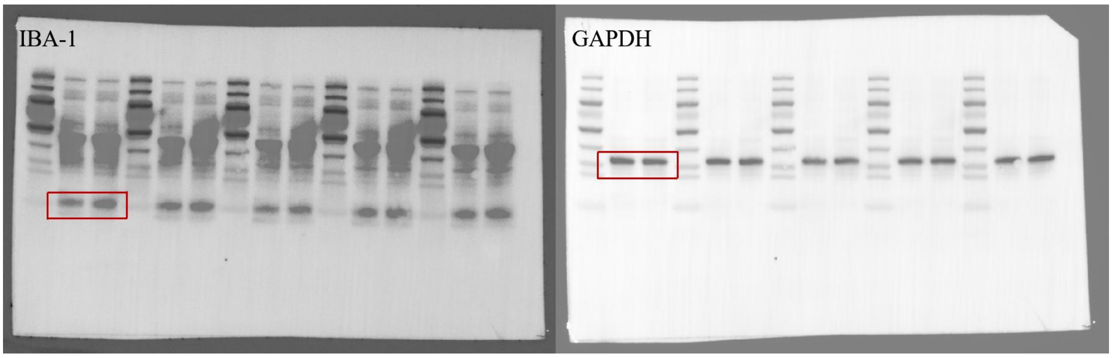

(B) GFAP

Full-length Western blot membrane showing GFAP and GAPDH expression in hippocampal samples from TD-FMT and ASD-FMT mice.

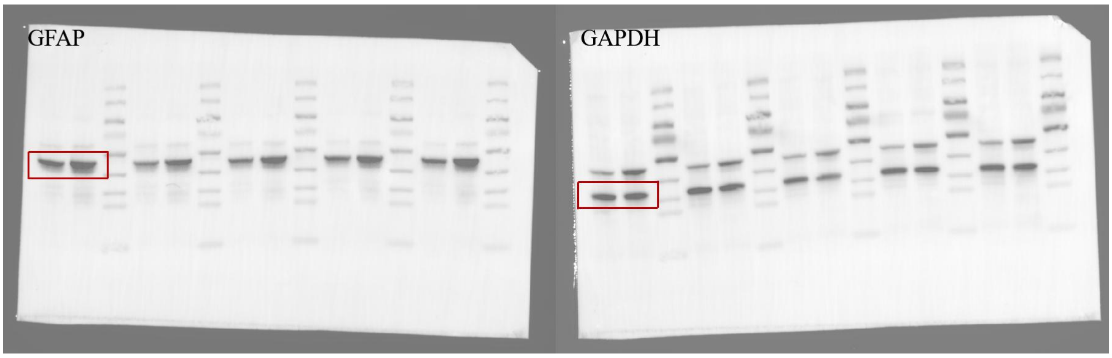

Supplement: Supplemental Information 7 [file peerj-14-20951-s007.pdf]
